# Environmental Persistence and Genotypic and Phenotypic Characterization of *Salmonella* Minnesota in Poultry Slaughterhouses

**DOI:** 10.3390/pathogens15030247

**Published:** 2026-02-26

**Authors:** Larissa Justino, Ana Angelita Sampaio Baptista, Rafael Humberto de Carvalho, Tiago Casella, Evelin Lurie Sano, João Vitor da Silva Costa, Arthur Roberto da Costa, Maísa Fabiana Menck-Costa, Maria Fernanda Marques Pilli, Ana Carolina Bergamo Benteo, Marielen de Souza, Alceu Kazuo Hirata, Carlos Adelino Dalle Mole, Rafael Mesalla Costalonga Andrade, Raphael Lucio Andreatti Filho, Alexandre Oba

**Affiliations:** 1 Laboratory of Animal Nutrition, Department of Zootechny, Agricultural Sciences Center, State University of Londrina, Londrina 86057-970, Brazil; 2 Laboratory of Avian Medicine, Department of Preventive Veterinary Medicine, Center for Agrarian Sciences, State University of Londrina, Londrina 86057-970, Brazil; 3 Animal Science Program, Center of Agrarian Sciences, State University of Londrina, Londrina 86057-970, Brazil; 4 Department of Dermatological, Infectious and Parasitic Diseases, Center for Microorganism Research, Faculty of Medicine of São José do Rio Preto, São José do Rio Preto 15090-000, Brazil; 5 Laboratory of Fish Bacteriology, Department of Preventive Veterinary Medicine, State University of Londrina, Londrina, 86057-970, Brazil; 6 Vetanco Brazil, Chapecó 89813-824, Brazil; 7 Laboratory of Ornithopathology, Department of the of Veterinary Clinic, Center for Animal Pathology, School of Veterinary Medicine and Animal Science (FMVZ), São Paulo State University (UNESP), Botucatu 18618-681, Brazil

**Keywords:** *Salmonella* Minnesota, poultry slaughterhouse, biofilm, antimicrobial resistance, virulence, PFGE

## Abstract

*Salmonella* Minnesota (SM) is considered an emerging serovar, adapted to the poultry production chain, frequently associated with antimicrobial resistance, biofilm formation, and environmental persistence. This study aimed to characterize SM isolates from a poultry slaughterhouse regarding phenotypic and genotypic profiles of antimicrobial resistance, biofilm-forming capacity, thermal tolerance, genotypic virulence profile, and clonal relatedness. Strains obtained from carcasses (*n* = 26), cecal contents (*n* = 25), and chiller water (*n* = 11) from the slaughterhouse were evaluated. A high frequency of resistance to β-lactams, multidrug-resistant phenotypes, and extended-spectrum β-lactamase-producing isolates were observed. All isolates harbored genes associated with virulence and biofilm formation (*invA*, *csgD*, and *adrA*). Biofilm formation was influenced by temperature, with greater intensity at refrigeration temperatures, especially on stainless steel surfaces. In thermal tolerance assays, a negative correlation between temperature and bacterial viability was observed. Genetically related lineages circulating among cecum, carcass, and slaughterhouse chiller water over time were observed. These findings indicate that the persistence of SM in poultry slaughterhouses is sustained by the interaction between antimicrobial resistance, adaptive capacity associated with biofilm formation, and the circulation of genetically related lineages, representing a relevant challenge for food safety and public health.

## 1. Introduction

*Salmonella* spp. is one of the main bacteria related to foodborne disease outbreaks, causing a significant impact on the economy and public health [1]. There are approximately 2650 serovars of *Salmonella* spp., and poultry products are among the main sources of pathogen transmission [2].

Variation in the prevalence of certain serovars relative to others has been observed [1]. Over the years, there has been an increase in the isolation of *Salmonella* Minnesota throughout the poultry production chain worldwide. In Brazil, it is considered one of the main serovars isolated in broiler farms [3,4], which is reflected in subsequent stages of the chain, being frequently found in carcasses and poultry by-products [5].

In slaughterhouses, cross-contamination by *Salmonella* spp. may occur at all stages of the slaughter line, such as evisceration, scalding, plucking machines, transportation, chilling, and the cut-up room [6]. The persistence of *Salmonella* spp. in slaughter plants may result in long-term contamination of products [7].

One of the main virulence factors that contribute to the survival of *Salmonella* spp. in the environment is the ability to form biofilms, which confers greater resistance to stressors and xenobiotics [1]. Biofilm formation is modulated by a set of intrinsic and extrinsic factors [8], among which temperature, the composition of the contact surface [9], pH, and nutrient availability stand out [10].

Biofilm formation in *Salmonella* spp. is mainly regulated by the *csgD* gene, which encodes the homonymous master regulator. This transcription factor activates the *csgBAC* operon, responsible for the synthesis of curli fimbriae, and induces the expression of the *adrA* gene, whose product regulates the activity of the *bcs* operons involved in cellulose biosynthesis [11]. Curli fimbriae, endowed with adhesive properties, act synergistically with the cellulose matrix, promoting cell aggregation, adhesion to surfaces, and, consequently, biofilm structuring [12].

The O antigen, which corresponds to the terminal polysaccharide portion of lipopolysaccharides (LPS) of the outer membrane, plays an essential role in protecting *Salmonella* spp. against stress conditions, such as desiccation, contributing to their persistence in the environment [12]. In addition to acting as a physicochemical barrier, LPS confers electrostatic charges to the bacterial surface, modulating interactions with substrates and influencing the initial stages of adhesion. Furthermore, the length of the LPS chain, defined by the extension of the O antigen, constitutes a critical factor in biofilm formation, since rough variants, characterized by shorter chains, show greater efficiency in initial adhesion and favor the development of more structured and stable biofilms [13].

Biofilm formation is directly associated with bacterial resistance [10]. This phenomenon results from the physicochemical barrier of the extracellular matrix, which presents a negative charge, polysaccharides that limit permeability, and enzymes capable of degrading antimicrobials, reducing their effective concentration [14]. In addition, the biofilm creates a microenvironment that favors horizontal transfer of resistance genes, contributing to the dissemination and maintenance of bacterial multidrug resistance [10,14].

*Salmonella* Minnesota isolates may carry multiple antimicrobial resistance genes [15,16]; moreover, their adaptive capacity and genetic plasticity [8] ensure persistence and constitute a significant risk to public health [16]. Brasão et al. [17] identified low genetic heterogeneity among *S.* Minnesota isolated from different industries, which were able to persist in the environment for years, demonstrating the presence of clones adapted to the poultry environment.

The presence of virulence genes in *S.* Minnesota isolated from the poultry chain is associated with its capacity for invasion, survival, and adaptation to the host. These genes encode factors that facilitate bacterial entry into cells, increase resistance to host defense mechanisms, and enhance pathogenic potential, contributing to the success of infection and persistence in the animal production environment [18].

The persistence of *S.* Minnesota in the Brazilian poultry environment has been associated with the megaplasmid pESM (plasmid for emergent *Salmonella* Minnesota), which carries genes conferring antimicrobial resistance to tetracycline (*tetA*) and broad-spectrum β-lactams (*bla*CMY-2), environmental tolerance to mercury (the *mer* operon), and the siderophore yersiniabactin (Yersinia high-pathogenicity island), related to iron uptake. This follows a pattern observed in other serovars, such as the presence of pESI (*S.* Infantis) and pSH-359.42 (*S.* Heidelberg), contributing to the emergent success of *S.* Minnesota in Brazil [19].

*Salmonella* Minnesota presents a set of virulence genes, high biofilm-forming capacity, broad phylogenetic diversity, and genetic plasticity, factors that confer to this serovar a marked adaptive potential and zoonotic character [8]. Thus, studies focused on its characterization may support the development of more effective prevention and control strategies, contributing to improved product quality and the assurance of food safety [20].

The persistence of *Salmonella* spp. isolates in slaughterhouses is configured as a multifactorial phenomenon. Studies demonstrate that the ability of *Salmonella* spp. to adhere to and form biofilms on surfaces such as stainless steel, under temperatures compatible with the slaughterhouse environment, contributes significantly to the permanence of the pathogen along the slaughter line [21,22]. Among the critical points of processing, the chilling tank stands out as an important source of contamination and dissemination, since positive batches can contaminate the immersion water and, subsequently, other carcasses [6,23].

In addition, multidrug-resistant *Salmonella* spp. isolates present along the slaughter line in slaughterhouses raise concern due to the risk of contamination for humans via food [24]. This scenario becomes even more critical in light of the detection of isolates exhibiting co-resistance to β-lactams and fluoroquinolones, classes of antimicrobials of critical importance for human medicine [25].

Therefore, the objective of this study was to characterize *Salmonella* Minnesota isolates obtained from slaughterhouse samples by evaluating the genotypic profiles of virulence and antimicrobial resistance, the phenotypic profiles of biofilm production and antimicrobial resistance, as well as thermal tolerance and clonal.

## 2. Materials and Methods

### 2.1. Sample Collection

The *Salmonella* spp. isolates used in this study are part of the bacterial collection of the Laboratory of Avian Medicine at the State University of Londrina (UEL) and were obtained from weekly samplings carried out at a commercial poultry slaughterhouse, between 2022 and 2023 (12 months). Initially, a total of 80 *Salmonella* spp. isolates were obtained.

From these, only isolates identified as *Salmonella* Minnesota were selected for the present study. For composition, samples from different sources (cecum, carcass, and slaughterhouse chiller water) were selected. Only one *Salmonella* spp. isolate per source (cecum, carcass, or slaughterhouse chiller water) per week was considered, randomly selected. At the end of the selection process, isolates were subjected to serotyping using the Check & Trace *Salmonella*^®^ method, resulting in 62 *Salmonella* Minnesota isolates from carcasses (*n* = 26), ceca (*n* = 25), and chilling water (*n* = 11).

### 2.2. Antimicrobial Susceptibility Testing and Phenotypic Profile of Extended-Spectrum β-Lactamase Production

Antimicrobial susceptibility testing was performed by the disk diffusion method and interpreted according to the Clinical & Laboratory Standards Institute [26]. Seven classes of antimicrobials were used: β-lactams: ampicillin (AMP—10 μg), amoxicillin–clavulanic acid (AMC—10/20 μg), cefepime (CPM—30 μg), ertapenem (ETP—10 μg), cefoxitin (FOX—30 μg), ceftriaxone (CRO—30 μg), ceftazidime (CAZ—30 μg), cefotaxime (CTX—30 μg), aztreonam (ATM—30 μg), and ceftiofur (XNL—30 μg); quinolones: ciprofloxacin (CP—5 μg) and enrofloxacin (ENR—5 μg), norfloxacin (NOR—10 μg); sulfonamides: sulfamethoxazole + trimethoprim (SXT—1.25/23.75 μg); tetracyclines: tetracycline (TET—30 μg); aminoglycosides: gentamicin (GEN—10 μg) and neomycin (N—30 mg); amphenicols: chloramphenicol (CL—30 μg); and fosfomycin: fosfomycin (FOS—200 μg). *E. coli* ATCC 25922 was used as a control. Multidrug resistance (MDR) was characterized as resistance to three or more classes of antimicrobials [27].

To evaluate extended-spectrum β-lactamase (ESBL) production, the double-disk synergy test was performed according to previous protocol [26].

### 2.3. Evaluation of Biofilm Formation on Polystyrene Plates

The biofilm formation assay in 96-well polystyrene microplates was performed according to Garcia et al. [28]. Briefly, the samples were cultured in Tryptic Soy Broth (TSB) (BD Difco™, Detroit, MI, USA) at 35 °C for 24 h and subsequently adjusted to a concentration of 10^8^ CFU/mL. Then, 250 µL aliquots were distributed in triplicate into the wells and incubated under the same conditions described above. After incubation, the wells were washed with phosphate-buffered saline (PBS; pH 7.2), fixed with methanol, and stained with 1% crystal violet for 15 min. Subsequently, new washing steps were performed, and the adhered biofilm was resuspended with 33% glacial acetic acid. The enteroaggregative Escherichia coli (EAEC) 042 strain was used as a positive control, while sterile TSB (BD Difco™, Detroit, MI, USA) was used as the negative control of the assay. Absorbance was measured at 630 nm using a microplate reader (Loccus^®^, Cotia, São Paulo, Brazil). The cutoff point (ODc) was defined as the mean optical density (OD) of the negative control plus three standard deviations. Isolates were classified as non-biofilm producers (OD ≤ ODc), weak producers (ODc < OD ≤ 2 ODc), moderate producers (2 ODc < OD ≤ 4 ODc), and strong biofilm producers (OD > 4 ODc).

### 2.4. Evaluation of Biofilm Formation on Stainless Steel Surface

Biofilm formation was evaluated on stainless steel (AISI 304), a material used in the manufacture of cooling equipment and processing surfaces in slaughterhouse cutting rooms. For the assays, stainless steel discs (1 cm in diameter) were used, previously cleaned and sterilized. The assay was performed according to De Oliveira et al. [29] with modifications. Initially, *Salmonella* Minnesota isolates were cultured in Brain Heart Infusion (BHI) broth (BD Difco™, Detroit, MI, USA) at 35 °C for 24 h. Subsequently, the cultures were diluted to 10^8^ CFU/mL, and 600 µL aliquots were distributed, in triplicate, into wells of polystyrene plates containing sterile stainless steel discs. The plates were incubated at 35, 16, and 12 °C for 96 h. After this period, the discs were carefully washed with sterile PBS and subjected to staining with crystal violet (0.1%). Subsequently, the stained biofilm was resuspended in 600 µL of glacial acetic acid (33%) and transferred to 96-well plates for spectrophotometric reading at 546 nm (Loccus^®^, Cotia, São Paulo, Brazil). *Salmonella* Typhimurium ATCC 14028 was used as a positive control. Classification of biofilm production capacity (non-producer, strong, moderate, or weak) was performed according to [30].

### 2.5. Detection of Virulence and Resistance Genes

Detection of virulence and resistance factors was performed using genomic DNA extracted from *Salmonella* Minnesota isolates with the PureLink Genomic DNA Mini Kit (Invitrogen, Waltham, MA, USA), according to the manufacturer’s instructions.

For virulence, primers previously described by Webber et al. [31] were used for the genes *invA*, *lpfA*, ag*fA*, *sefA*, *avrA*, *spaN*, *tolC*, *sipA*, and *luxS*, following the protocol proposed by the same authors. Amplification of the *csgD* gene was conducted according to Chen et al. [32], whereas the *adrA* gene was analyzed according to a protocol developed in this study (Table A1).

PCR reactions were prepared in a final volume of 25 µL, containing 12.5 µL of GoTaq^®^ Green Master Mix (Promega, Madison, WI, USA), 1 µL of each primer (1 µM), 2 µL of template DNA, and 8.5 µL of ultrapure water. Cycling conditions adopted for the genes described by Webber et al. [31] and Chen et al. [32] followed the respective original protocols, whereas amplification of the *adrA* gene was performed according to De Oliveira et al. [29]. The amplified products were subjected to electrophoresis on 1.5% agarose gels stained with GelRed™ (Biotium, Fremont, CA, USA) and visualized using a transilluminator (Loccus^®^, Cotia, São Paulo, Brazil). The *Salmonella* Typhimurium ATCC 14028 strain was used as a positive control for the reaction.

Additionally, genes associated with resistance to β-lactams (*bla*CTX-M-1, *bla*CTX-M-2, *bla*CTX-M-8, *bla*CTX-M-9, and *bla*CTX-M-25) and to fosfomycin (*fosA3*) were investigated as described by Menck-Costa et al. [33], as well as genes related to quinolone resistance (*qnrA*, *qnrB*, and *qnrS*), according to Robicsek et al. [34].

### 2.6. Thermal Tolerance

Thermal tolerance evaluation was conducted with *Salmonella* Minnesota isolates exposed to different combinations of temperature and time, representative of conditions observed at critical stages of industrial poultry processing. The isolates were subjected to 4 °C for 30 min, simulating the cooling process; 10 °C for 30 min, corresponding to conditions in cutting and deboning rooms [35]; 37 °C for 30 min, the optimal temperature for bacterial growth; 50 °C for 3 min, representing the lower range of the scalding process; and 65 °C for 3 min, corresponding to the maximum scalding temperature. For the assay, samples were previously cultured in BHI broth (BD Difco™, Detroit, MI, USA) at 37 °C for 24 h. Subsequently, the cultures were diluted in PBS for initial bacterial quantification (CFU/mL) prior to thermal exposure. Samples were exposed to the established experimental conditions in a dry bath (Loccus^®^, Cotia, São Paulo, Brazil). Thereafter, the suspensions were diluted in PBS and plated on MacConkey agar at 37 °C for 24 h, and bacterial growth was evaluated to estimate survival after each treatment. *Salmonella* Enteritidis ATCC 13075 was used as a positive control.

### 2.7. Pulsed-Field Gel Electrophoresis (PFGE)

The isolates were typed by DNA restriction with *XbaI* (Thermo Scientific, Waltham, MA, USA) followed by a pulsed-field gel electrophoresis (PFGE) using the CHEF-DR II system (BioRad, Hercules, CA, USA), as described [36]. Few modifications were applied: electrophoresis = 19 h, initial and final switch times = 2.2 s and 63.8 s. The dendrogram was constructed and clustered using BioNumerics™ 7.6 (Applied Maths-bioMérieux, Sint-Martens-Latem, Belgium), the Dice similarity coefficient, and the UPGMA method.

Isolates sharing a minimum similarity of 90% were considered genetically closely related and were assigned to the same pulsotype, whereas similarity values ≥ 80% were interpreted as indicative of isolates belonging to the same genetic lineage. The interpretation criteria were based on previous studies investigating the persistence and dissemination of *Salmonella* spp. in poultry slaughterhouses [37].

### 2.8. Statistical Analysis

All analyses considered each *Salmonella* Minnesota isolate as the experimental unit. Isolates originated from cecum, carcass, and slaughterhouse chiller water. When appropriate, temporal variability related to the sampling process was accounted for by including month of sampling as a random effect to avoid pseudoreplication.

Variables were analyzed according to their distribution and data structure. Binary outcomes were analyzed using logistic regression models, ordinal outcomes using ordinal regression models, and continuous outcomes using linear models. Mixed-effects models were applied when hierarchical or repeated-measures structures were present, with isolate included as a random effect when the same isolate contributed multiple observations.

Phenotypic antimicrobial resistance, biofilm category frequencies, and the presence of virulence and resistance genes were analyzed as binary or ordinal outcomes, with sampling origin included as a fixed effect. Outcomes with zero variance were not subjected to inferential analysis. When overall effects were significant, pairwise comparisons were performed using model-based estimated marginal means with adjustment for multiple testing.

Thermal tolerance was evaluated using bacterial counts expressed as log_10_(CFU/mL) under a repeated-measures framework, including sampling origin and thermal condition as fixed effects and isolate as a random intercept. Pairwise comparisons were conducted when appropriate using adjusted post hoc tests.

Control of multiple comparisons was applied using the Benjamini–Hochberg false discovery rate procedure when relevant. Statistical significance was set at α = 0.05. All analyses were performed using Statistica 10.0 (StatSoft Inc., Tulsa, OK, USA).

## 3. Results

### 3.1. Antimicrobial Susceptibility Profile and Extended-Spectrum β-Lactamase Production

The phenotypic antimicrobial resistance profile of *Salmonella* Minnesota isolates obtained from carcasses, cecal contents, and slaughterhouse chiller water is presented in Table 1.

A high frequency of resistance to β-lactams was observed, especially to third-generation cephalosporins (cefotaxime, ceftazidime, ceftriaxone, and ceftiofur). A statistically significant difference was observed only for the antimicrobial ceftiofur (*p* = 0.040), which showed a higher frequency of resistance among isolates from ceca (96.0%; 24/25), differing significantly from those obtained from carcasses (69.23%; 18/26), while isolates from chiller water showed intermediate resistance values (72.73%; 8/11) (Table 1).

Regarding second- and fourth-generation cephalosporins, no statistically significant difference was observed among the matrices evaluated (*p* = 0.6310 and *p* = 0.8070, respectively), with frequencies of 36.36% and 69.23% of isolates resistant to these classes being observed. Regarding fosfomycin, low resistance rates were observed, restricted to 11.5% (3/26) of isolates from carcasses, with no resistant isolates detected in cecal and slaughterhouse chiller water samples (*p* = 0.116). In contrast, resistance to fluoroquinolones, ciprofloxacin resistance was detected in 65.38% (7/11) of isolates from carcasses, 52% (15/25) of isolates from ceca, and 54.55% of isolates from slaughterhouse chiller water. Resistance to enrofloxacin was similar among origins, with 63.58% in carcasses, 68% in ceca, and 63.64% (7/11) in slaughterhouse chiller water (*p* = 0.964). Regarding norfloxacin, resistant *Salmonella* Minnesota isolates were observed only in carcass samples, accounting for 7.96% (2/26).

**Figure 1:**
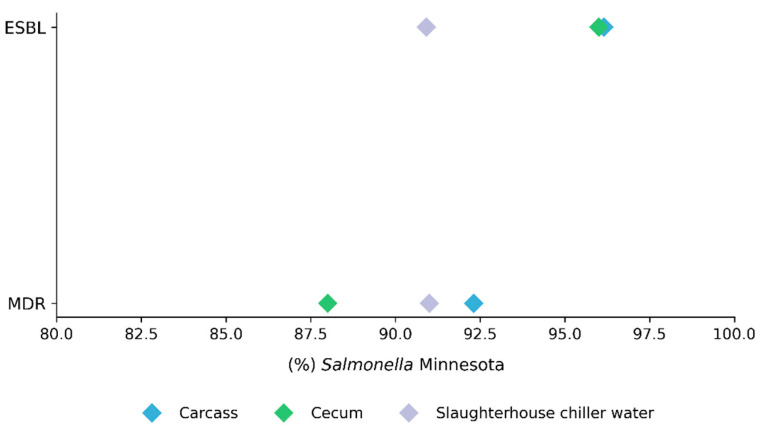
Distribution of *Salmonella* Minnesota isolates classified as multidrug-resistant (MDR) and extended-spectrum β-lactamase (ESBL) producers according to sampling origin (carcass, cecum, and slaughterhouse chiller water). Values represent the percentage of isolates within each origin. Comparisons among origins were performed using binomial generalized linear mixed-effects models, with origin as a fixed effect and month of sampling as a random effect. No significant differences were detected for MDR (*p* = 0.876) or ESBL production (*p* = 0.777).

Most *S.* Minnesota isolates exhibited a multidrug-resistant (MDR) phenotype, with 92.31% (24/26), 88% (22/25), and 91% (10/11) recovered from carcasses, ceca, and slaughterhouse chiller water, respectively; however, no significant differences were observed among origins (*p* = 0.876). A similar result was observed for ESBL enzyme production (*p* = 0.778), with frequencies of 96.1% (25/26), 96% (24/25), and 90.9% (10/11) in the same samples (Figure 1).

### 3.2. Biofilm Formation on Polystyrene Plates

The biofilm-forming capacity of *S.* Minnesota isolates on polystyrene plates showed a significant difference among origins (carcass, cecum, and slaughterhouse chiller water) only for the weak biofilm category (Table 2). In this category, isolates from chiller water showed a higher frequency, differing significantly from carcass isolates, whereas isolates originating from the cecum showed intermediate values.

Overall, the results indicate that although most isolates exhibited moderate or strong biofilm-forming phenotypes, only the frequency of weak biofilm formation differed significantly among origins.

### 3.3. Biofilm Formation on Stainless Steel Surface

The biofilm production capacity of *Salmonella* Minnesota isolates on stainless steel was evaluated, and a higher proportion of non-adherent isolates was observed at 35 °C (38.71%; 24/62), which was significantly higher than that observed at 12 °C (11.29%; 7/62) and 16 °C (9.68%; 6/62) (*p* = 0.000) (Table 3).

**Figure 2:**
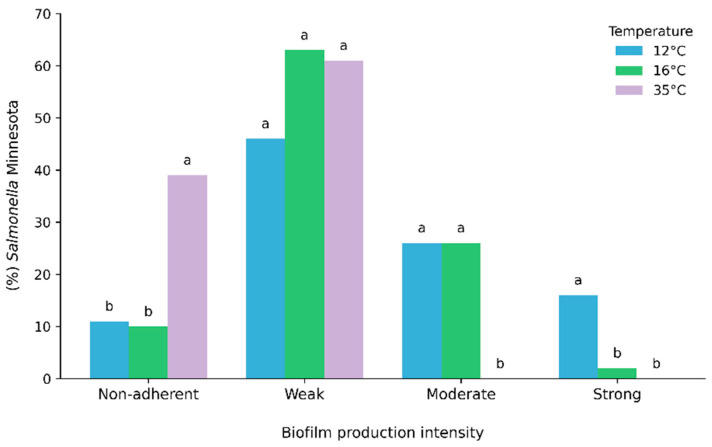
Biofilm production intensity of *Salmonella* Minnesota isolates on stainless steel at 12, 16, and 35 °C. Bars represent the percentage of isolates classified as non-adherent, weak, moderate, or strong biofilm producers at each temperature. Differences among temperatures within each bio-film category were evaluated using an ordinal mixed-effects model including temperature, origin, and temperature × origin as fixed effects and isolate as a random intercept. Different lowercase letters indicate significant pairwise differences among temperatures within the same category after multiplicity adjustment (*p* < 0.05).

Moderate biofilm production was notable at 12 °C (25.81%; 16/62) and 16 °C (25.81%; 16/62), with absence at 35 °C (0%; 0/62) (*p* = 0.224). Strong biofilm formation was more frequent at 12 °C (16.13%; 10/62), being significantly higher than that observed at 16 °C (1.61%; 1/62) and 35 °C (0%; 0/62) (*p* = 0.001) (Table 3; Figure 2).

No significant differences were observed regarding sample origin (carcass, cecum, or slaughterhouse chiller water) nor for the origin × temperature interaction (*p* > 0.05), indicating that biofilm formation depends on temperature and is independent of origin (Table 3).

### 3.4. Virulence and Resistance Genes

Detection of virulence genes in *Salmonella* Minnesota demonstrated a high prevalence of genes associated with cell invasion and biofilm formation (Table 4).

A high frequency of the genes *adrA*, *csgD*, *invA*, *agfA*, *sipA*, *spaN*, *tolC*, and *avrA* was observed among *Salmonella* Minnesota isolates, regardless of the source (carcass, cecum, or chilling water). These findings indicate a wide distribution of these virulence genes among *Salmonella* Minnesota isolates from different sample matrices (Table 4). In contrast, the *luxS* gene, associated with the quorum sensing system, showed low prevalence, being detected in 8.06% (5/26) of carcass isolates and in 36% (9/25) of cecal isolates, and was not identified in samples of slaughterhouse chilling water (*p* = 0.051).

The distribution of resistance genes did not show significant differences among the different sample origins (carcass, cecum, and slaughterhouse chiller water), as shown in Table 5.

Regarding of quinolone and cephalosporin associated resistance in *Salmonella* Minnesota isolates revealed that the *qnrB* gene was the most prevalent, detected in 69.23% (18/26) of carcass isolates, 68% (17/25) of cecal isolates, and 54.55% (6/11) of slaughterhouse chiller water isolates, with no significant differences among origins (*p* = 0.678) (Table 5). Among the CTX-M family genes, which confer resistance to β-lactam antimicrobials, *bla*CTX-M-2 showed the highest frequency, being present in 38.46% (10/26) of carcass isolates, 52% (13/25) of cecal isolates, and 45.46% (5/11) of slaughterhouse chiller water isolates, with no significant differences between origins (*p* = 0.636).

### 3.5. Thermal Tolerance of Salmonella Minnesota Isolates

Thermal tolerance analysis of *Salmonella* Minnesota isolates demonstrated temperature-dependent variations (Table 6). At 4 °C, bacterial viability was significantly lower (*p* = 0.0342) in carcass isolates (9.084 log_10_ CFU/mL) compared with slaughterhouse chiller water isolates (9.247 log_10_ CFU/mL), while cecal isolates showed intermediate values (9.139 log_10_ CFU/mL).

At 65 °C, a marked reduction in bacterial viability was observed, with isolates from cecum showing the highest mean value (2.162 log_10_ CFU/mL), which was significantly higher (*p* = 0.0419) than that observed in carcasses (1.647 log CFU/mL), while slaughterhouse chiller water presented an intermediate value (1.987 log_10_ CFU/mL).

**Figure 3:**
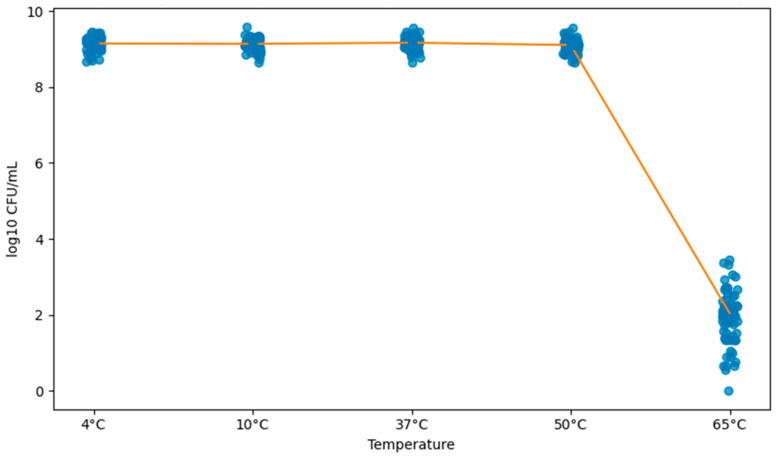
Exploratory relationship between temperature and viability of *Salmonella* Minnesota isolates expressed as log_10_ CFU/mL. Dots represent individual observations obtained from isolates recovered from carcasses, cecum, and slaughterhouse chiller water. Mean values ± 95% confidence intervals are overlaid for each temperature–time condition. The curve represents a locally estimated scatterplot smoothing (LOESS) and is shown for descriptive purposes only, illustrating the overall trend and the marked reduction in bacterial viability at 65 °C. Formal statistical inference and pairwise comparisons among origins within each condition were performed using linear mixed-effects models and are presented in Table 6.

Figure 3 shows that *Salmonella* Minnesota survival was inversely proportional to increasing temperature, with a marked decline in bacterial counts above 50 °C.

Figure 3 shows a segmented regression model was fitted to describe the relationship between temperature and bacterial viability (log_10_ CFU/mL), accounting for repeated observations per isolate using cluster-robust standard errors and adjusting for sampling origin. The estimated breakpoint was 49.8 °C (≈50 °C). Below the breakpoint, viability showed a minimal positive slope (β = 0.00133 log_10_ CFU/mL per °C; 95% CI: 0.00048–0.00218; *p* = 0.002). Above the breakpoint, viability decreased sharply (β = −0.47860 log_10_ CFU/mL per °C; 95% CI: −0.49098 to −0.46622; *p* < 0.001), consistent with a marked loss of culturability at higher temperatures. Compared with carcass isolates, cecum isolates showed slightly higher counts (β = 0.1366; 95% CI: 0.0414–0.2317; *p* = 0.004), while chiller-water isolates presented a similar tendency (β = 0.1408; 95% CI: −0.0085–0.2901; *p* = 0.064).

### 3.6. Genetic Relatedness by Pulsed-Field Gel Electrophoresis (PFGE)

**Figure 4:**
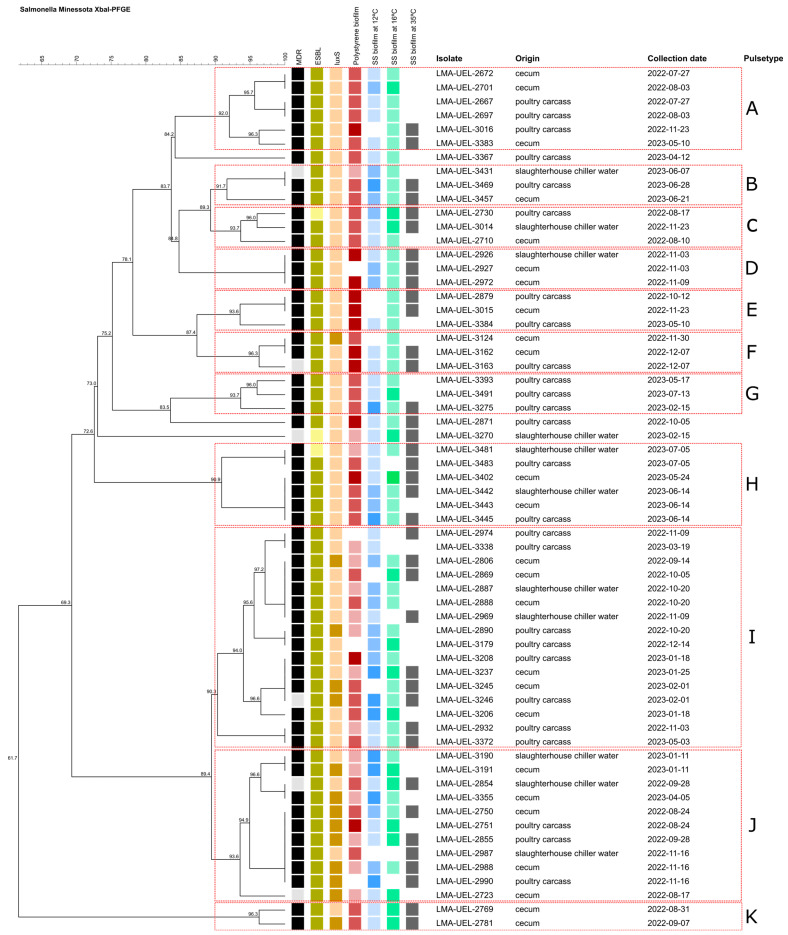
Dendrogram of *Salmonella* Minnesota isolates recovered from cecum, poultry carcasses, and slaughterhouse chiller water, generated by XbaI-PFGE. A similarity cut-off of 80% was applied to define major genetic lineages, while isolates sharing ≥90% similarity were assigned to the same pulsetype. Subclusters presenting ≥95% similarity were classified as possibly clonal. Pulsetipes (A to K) are indicated by dashed red lines. Multidrug-resistant (MDR): light-gray square = negative, black square = positive. Extended-spectrum β-lactamase (ESBL): light-yellow square = negative; dark-yellow square = positive. luxS gene: light salmon square = negative; dark salmon square = positive. Polystyrene biofilm: white square = non-producer; light-red square = weak producer; medium-red square = moderate producer, dark-red square = strong producer. Stainless steel biofilm (SS biofilm) at 12 °C: white square = non-producer, light-blue square = weak producer, medium-blue square = moderate producer, dark-blue square = strong producer. Stainless steel biofilm (SS biofilm) at 16 °C: white square = non-producer, light-green square = weak producer, medium-green square = moderate producer, dark-green square = strong producer. Stainless steel biofilm (SS biofilm) at 35 °C: white square = non-producer; gray square = weak producer.

*XbaI*-PFGE macrorestriction analysis revealed high genetic similarity among *Salmonella* Minnesota isolates obtained from the different sample origins. The dendrogram shows the formation of multiple clusters, with similarity coefficients predominantly ≥80%, indicating the circulation of genetically related lineages, and several clusters presenting values ≥ 90%, corresponding to genetically closely related isolates assigned to the same pulsotype (Figure 4).

The *Salmonella* Minnesota isolates showed high genetic heterogeneity, being distributed into 11 pulsotypes (A–K). It was observed that isolates obtained from the cecum frequently clustered with those from carcasses and chiller water, especially in pulsotypes A, D, and F, suggesting that the birds already arrived positive at the slaughterhouse and that the intestinal tract acted as the primary source of dissemination, with subsequent contamination of the carcass and the processing environment (Figure 4).

The simultaneous recovery of *S*. Minnesota from the cecum and other matrices (chiller water or carcass), on the same sampling date, in distinct pulsotypes (A, D, F, H, I, and J), demonstrates the coexistence of multiple genetic profiles throughout the process. It was observed that isolates from chiller water were distributed into four pulsotypes (D, H, I, and J), reinforcing the role of the chiller in the dynamics of redistribution and maintenance of different *S*. Minnesota pulsotypes in the processing environment.

The majority of *S*. Minnesota isolates from chiller water (82%) showed the ability to form biofilm on stainless steel at 16 °C, being distributed among different pulsotypes (B, C, D, H, I, and J). In contrast, 96% of the isolates originating from carcasses formed biofilm on stainless steel at 12 °C, a temperature similar to that observed in the cutting room.

In the same month, *S*. Minnesota isolates of pulsotype B were obtained from the cecum, carcass, and chiller water, which showed high genetic similarity. In pulsotypes B and C, recurrence of isolates over time was observed. Only pulsotype B showed positivity in three consecutive weeks, whereas in pulsotype C, chiller water again showed positivity three months after the recovery of isolates from the cecum and carcass.

Additionally, pulsotypes A, E, I, and J were identified in the years 2022 and 2023, with an interval of 6–10 months between recoveries.

## 4. Discussion

*Salmonella* Minnesota shows high prevalence [3] and a strong ability to persist in slaughterhouse environments [6], which is particularly concerning when associated with the recurrent detection of multidrug-resistant profiles in this serovar [15,16]. Poultry carcasses represent a potential source of human infection and may also act as reservoirs of antimicrobial resistance genes, thereby facilitating their dissemination to other microorganisms [38].

In the present study, a high level of phenotypic resistance to β-lactams was observed, particularly to third-generation cephalosporins, accompanied by a high frequency of ESBL-producing isolates. This correspondence between phenotype and genotype suggests that the detected resistance is associated with the presence of genes related to β-lactamase production, which are frequently located on plasmids. These findings reinforce the role of *Salmonella* Minnesota as an important reservoir of clinically relevant genetic determinants of resistance and highlight the potential for dissemination of these genes in slaughterhouse environments through horizontal gene transfer mechanisms [39].

Resistance to ciprofloxacin and enrofloxacin highlights the persistence of quinolone resistance, a class of antimicrobials considered critically important within the One Health context due to their concomitant use in veterinary and human medicine [16]. In addition, the higher frequency of resistance to ceftiofur in cecal isolates (*p* = 0.040) suggests that the intestinal tract of poultry may function as a reservoir and amplification site for resistant strains. Although ceftiofur is a third-generation cephalosporin approved exclusively for veterinary use [40], its use is of particular concern because of the potential for co-selection and cross-resistance with cephalosporins of critical importance in human medicine [41,42].

Genes of the qnr family encode proteins that bind to bacterial topoisomerases, protecting these target enzymes and reducing the efficacy of fluoroquinolones by interfering with the formation of the DNA–topoisomerase complex. In parallel, the *aac(6′)-Ib-cr* gene acts by structurally modifying certain fluoroquinolones through acetylation of the piperazinyl group, thereby decreasing their antimicrobial activity. These mechanisms, which are frequently associated with mobile genetic elements such as plasmids, promote both the horizontal dissemination and persistence of quinolone resistance among different bacterial populations [43]. In the present study, a high frequency of the *qnrB* gene was observed in isolates from different sources, with no significant variation among matrices, indicating that quinolone resistance in *S.* Minnesota is predominantly associated with plasmid-mediated mechanisms, as previously described for *Salmonella* spp. [44].

Partially corroborating the findings of the present study, Yan Lu et al. [45] reported high rates of quinolone resistance in *Salmonella* Indiana isolates from slaughterhouses, with frequencies of 59.0%, 79.5%, and 80.2% for enrofloxacin, norfloxacin, and ciprofloxacin, respectively. However, unlike the results observed in our study, the authors did not detect genes of the qnr family (*qnrA*, *qnrB*, and *qnrS*), identifying only the *aac(6′)-Ib-cr* gene and mutations in the *gyrA* gene, both associated with quinolone resistance. These findings indicate the coexistence of chromosomal and plasmid-mediated resistance mechanisms and suggest that the determinants involved may act independently or in combination, possibly being located on the same plasmid and playing a relevant role in plasmid-mediated resistance.

The high frequency of multidrug-resistant *S*. Minnesota isolates, observed regardless of sample origin, suggests that these profiles are widely disseminated throughout the slaughter chain [46], possibly sustained by mechanisms that favor environmental persistence. Previous studies have shown that this serovar exhibits a high capacity for adaptation to industrial environments, which is associated with both antimicrobial resistance and persistence on slaughterhouse surfaces and equipment [6,16,17,18,47,48].

Bacterial persistence may be further enhanced by the occurrence of cross-resistance between antimicrobials and biocidal agents, promoting the selection of multidrug-resistant and ESBL-producing phenotypes, as well as reducing the effectiveness of disinfectants used in the food industry. This phenomenon is associated with both genotypic and phenotypic adaptations, including the overexpression of efflux pumps, alterations in the cell membrane, increased production of β-lactamases and antioxidant enzymes, and the co-localization of antimicrobial- and biocide-resistance genes on mobile genetic elements, thereby facilitating horizontal gene transfer [49,50].

In this context, biofilm production by bacteria may drive the cross-resistance process by promoting the acquisition and maintenance of resistance genes to antimicrobials and biocidal agents [50], as well as increasing the potential for dissemination of multidrug-resistant profiles [14,51]. The association between multidrug resistance and biofilm formation observed in this study is consistent with previous findings in poultry-derived *Salmonella* spp., in which persistent isolates tend to exhibit an enhanced ability to survive under conditions of environmental stress [8,14,19,52].

In addition, the concept of bacterial recalcitrance provides a relevant theoretical framework for the interpretation of these results. Unlike classical genetic resistance, recalcitrance encompasses tolerance and persistence phenotypes that enable bacterial survival under antimicrobial pressure without necessarily involving stable genetic alterations [53]. Within biofilms, limited antimicrobial diffusion through the extracellular polymeric matrix and metabolic alterations favor these phenotypes, contributing to the persistence of *Salmonella* spp. in slaughterhouse environments, even in the presence of sanitation and control protocols [12,14,54,55].

In this context, phenotypically susceptible bacteria organized in biofilms may exhibit high tolerance to β-lactams through the phenomenon of cooperative resistance, in which collective protection is mediated by resistant bacteria via the local activity of β-lactamases [56]. In slaughterhouses, the persistence of biofilm-producing bacteria harboring resistance genes compromises the effectiveness of cleaning and disinfection procedures, promotes pathogen persistence on surfaces and equipment, and increases the risk of recontamination of the final product, with direct implications for food safety and public health [57]. This mechanism may explain, at least in part, the recurrent detection of multidrug-resistant isolates throughout the slaughter chain observed in this study.

In the present study, biofilm production on stainless steel by *S*. Minnesota isolates demonstrated a significant influence of temperature on biofilm phenotypes. A temperature of 12 °C favored the occurrence of strong and moderate biofilm-forming phenotypes. At 16 °C, similar proportions of moderate phenotypes were observed; however, there was a marked reduction in the frequency of isolates classified as strong biofilm producers. These findings reinforce that temperatures compatible with industrial refrigeration environments not only allow the survival of *Salmonella* spp. but may also promote the formation of structured biofilms, representing a significant risk for final product contamination [29].

The literature demonstrates that temperature is a determining factor in biofilm formation by *Salmonella* spp., as some strains activate specific molecular mechanisms under distinct thermal conditions, resulting in differential responses in adhesion and extracellular matrix production [58]. Vice et al. [59] demonstrated that at 15 °C, changes occur in the architecture and composition of the biofilm extracellular matrix, leading to the formation of more structured biofilms with matrices rich in extracellular polymeric substances (EPS). These characteristics favor cellular adhesion, bacterial persistence, and increased tolerance to hygiene procedures applied in industrial environments.

Kim et al. [60] demonstrated that *Salmonella* Typhimurium incubated at moderately low temperatures exhibits increased production of fimbriae, cellulose, and extracellular polymeric substances, resulting in more structured and stable biofilms compared with those formed at temperatures close to the optimal for bacterial growth. These findings are consistent with our results, in which incubation at 35 °C promoted a predominance of non-adherent phenotypes, indicating that although this temperature is near the optimal for bacterial growth, it does not favor the maintenance of biofilm structures on stainless steel. However, these results differ from those reported by De Oliveira et al. (2014) [29], who observed greater biofilm production on stainless steel at 35 °C, predominantly with a weak biofilm profile.

These discrepancies may be attributed to differences among serovars or to the genetic background of the evaluated isolates. In addition, they reinforce findings reported in the literature describing biofilm formation as an adaptive mechanism in response to environmental stress conditions, particularly in food-processing environments, where lower temperatures may favor bacterial persistence [58,61,62].

The greater capacity for biofilm formation at refrigeration temperatures observed in this study has direct implications for food safety, as industrial strategies based exclusively on cooling may be insufficient to eliminate *S*. Minnesota adhered to contact surfaces. This adaptive behavior reinforces the need for rigorous sanitation protocols, particularly for equipment operated at low temperatures, in order to reduce the risk of cross-contamination throughout the poultry production chain [63].

Biofilm production on polystyrene plates showed a significant difference only for the weak biofilm profile, with a higher frequency among isolates derived from chiller water. Regarding production intensity, a predominance of moderately producing phenotypes was observed regardless of sample origin, although no statistically significant difference was detected. The high hydrophobicity of polystyrene favors initial interactions with bacterial cell wall components and with fractions of extracellular polymeric substances, reducing interfacial energy and facilitating adhesion [64]. In addition, the presence of organic matter in chiller water may contribute to the formation of a conditioning film, further enhancing this process [65].

These findings are consistent with studies demonstrating a greater biofilm-forming capacity of *Salmonella* spp. on polystyrene surfaces compared with stainless steel, reinforcing the value of this material as an experimental tool for understanding the initial mechanisms of adhesion and biofilm maturation [58,66]. Chia et al. (2009) [21] reported that adhesion is associated with genetic composition, serovar, and strain, as well as with the presence of specific genes and the physicochemical properties of surfaces, indicating that *Salmonella* spp. may employ distinct mechanisms depending on the material, thereby highlighting the multifactorial nature of the adhesion process.

Considering that all *S*. Minnesota isolates in this study, regardless of origin, tested positive for the *adrA* and *csgD* genes, and that temperature significantly influenced the phenotypic analysis of biofilm formation on stainless steel surfaces, it is possible to infer an effect of temperature on the expression of these genes. However, it should be emphasized that the present study did not employ specific techniques to assess gene expression of virulence factors, which limits this inference to an indirect association based on phenotypic evidence.

Studies have shown that, in mature *Salmonella* spp. biofilms, curli fimbriae and cellulose constitute the main structural components of the extracellular matrix, with their expression coordinated by the regulator CsgD, which stimulates transcription of the *adrA* gene and activates curli and cellulose production at the post-transcriptional level [67,68]. Expression of the *csgD* gene is modulated by environmental signals such as temperature, nutrient availability, oxygen, osmolarity, and pH [11,68]. Temperatures above 32 °C and high nutrient availability repress this regulatory pathway, resulting in reduced biofilm formation [69]. In addition, the rdar morphotype (red, dry, and rough), characterized by the concomitant expression of cellulose and curli fimbriae, is predominantly observed at temperatures below 28 °C [70], highlighting the role of temperature in csgD regulation.

In addition, other genes are involved in biofilm formation [71], such as *agfA* and *luxS*, which were investigated in this study. The *agfA* gene showed a high frequency across all sample origins, whereas *luxS* was absent in isolates from chiller water and occurred more frequently in cecal isolates, suggesting a closer association of quorum sensing with the intestinal environment of poultry. In this context, high bacterial density favors communication mediated by autoinducers, contributing to the regulation of gene expression and bacterial persistence, as described by Dula et al. [9] as a relevant adaptive advantage. These findings partially corroborate the results reported by Melo et al. [8], who observed the concomitant presence of *agfA* and *luxS* in 75% of *Salmonella* Minnesota isolates, and further reinforce the genetic potential of these strains for biofilm formation.

Another relevant aspect of CsgD-mediated regulation is its role in bet-hedging, an adaptive strategy based on heterogeneous expression of this regulator within the bacterial population [72]. This mechanism results in the coexistence of physiologically distinct subpopulations, including planktonic cells, characterized by higher metabolic activity and increased host invasion potential, and aggregated cells, which are associated with biofilm formation and greater tolerance to environmental stresses. This functional differentiation constitutes an efficient long-term survival strategy for enteric pathogens that spend a substantial part of their life cycle in the environment [72].

In this context, the high frequency of genes associated with invasion and infection observed in the present study reinforces the importance of the planktonic state for the virulence of *Salmonella* Minnesota. The universal detection of the *invA* gene, a classical marker of cellular invasion [73], together with the high frequency of the *sipA*, *spaN*, *avrA*, and *tolC* genes, regardless of sample origin (carcass, cecum, and chiller water) and without statistically significant differences among origins (*p* > 0.05), highlights the high pathogenic and adaptive potential of this serovar [31,74,75]. These findings suggest that, although biofilm formation favors environmental persistence, the maintenance of a robust repertoire of virulence genes ensures infectious capacity when cells return to the planktonic state, thereby contributing to the emergence and persistence of *Salmonella* Minnesota throughout the production chain.

Environmental resistance is a relevant factor for bacterial persistence throughout the slaughter chain. In this study, temperature variations differentially influenced microbial counts at distinct points along the slaughter line, indicating niche-specific responses. A moderate and statistically significant negative correlation was observed between temperature and logarithmic counts (r = −0.7061; *p* < 0.0001), indicating a progressive reduction in the bacterial population with increasing temperature. The coefficient of determination (r^2^ = 0.4985) indicates that approximately 50% of the observed variability can be attributed to temperature, highlighting its central—although not exclusive—role in bacterial survival dynamics.

Under refrigeration conditions (4 °C), significant differences were observed among the sampled niches, with higher microbial counts in chiller water compared with carcasses (*p* = 0.0342). This result reinforces the role of the chiller as a critical point for microbial accumulation and redistribution, as previously described [6]. The presence of residual water, combined with the accumulation of organic matter and inadequate water renewal, favors the maintenance and dissemination of microorganisms in this environment [63].

Under exposure to elevated temperatures, specifically at 65 °C, a marked reduction in microbial counts was observed, with significant differences among the sampled points (*p* = 0.0419), indicating effective, although non-uniform, thermal inactivation. Carcasses exhibited lower counts compared with cecal samples, with no difference relative to chiller water. These findings partially align with studies reporting a pronounced reduction in *Salmonella* at high temperatures, with residual survival depending on the matrix and exposure time [76,77].

The high survival capacity of *S*. Minnesota under different environmental stresses, associated with biofilm formation and persistence at critical processing points such as chiller water, suggests that these phenotypes may be related to the maintenance of genetically related lineages over time. In this context, PFGE macrorestriction analysis was employed to investigate the population structure, as well as the dissemination and persistence patterns of this serovar along the poultry slaughter chain [16].

The PFGE profiles revealed high genetic heterogeneity among *S.* Minnesota isolates, indicating the simultaneous circulation of multiple lineages within the slaughterhouse environment. Nevertheless, the recurrence of genetically related pulsotypes over time and across different sample matrices demonstrates that, despite this diversity, certain clones exhibit a greater ability to persist and disseminate along the slaughtering process. As highlighted by Mughini-Gras et al. [78], the identification of genetically related clusters provides relevant epidemiological insights for source tracking and for understanding the circulation of *Salmonella* spp., even in the absence of completely indistinguishable profiles.

This pattern is consistent with both the environmental persistence of the microorganism and its continuous circulation among different matrices of the production process, including the cecum, carcass, and chiller water [4,37], as well as with the recurrent reintroduction of lineages adapted to poultry processing conditions [37,79].

In our study, the co-clustering of isolates from the cecum, carcasses, and chiller water particularly among shared pulsotypes—indicates that birds likely entered the slaughterhouse already positive, with the intestinal tract serving as the primary initial source of *Salmonella* spp. dissemination throughout processing. This finding is consistent with Zeng et al. [6], who demonstrated that the prior slaughter of contaminated flocks compromises the effectiveness of cleaning and disinfection procedures, thereby favoring pathogen persistence in the industrial environment. Under these conditions, *Salmonella* spp. can remain on surfaces, equipment, and in process water, promoting cross-contamination of carcasses and subsequent flocks during critical stages of the slaughter line.

Rasschaert et al. [80] demonstrated that certain *Salmonella* spp. strains exhibit a remarkable ability to survive in slaughterhouse environments, remaining viable even after the application of routine cleaning and disinfection procedures, which contributes to their persistence and dissemination throughout processing.

The recurrent occurrence of mixed clusters involving isolates from the cecum, carcasses, and chiller water reinforces the role of the avian intestinal tract as an important source for the introduction of *S*. Minnesota, followed by its dissemination along the slaughter line [81]. Conversely, the detection of genetically indistinguishable isolates in different matrices on the same sampling date, observed across several pulsotypes, is indicative of sporadic cross-contamination events, generally associated with temporary failures in hygienic–sanitary controls [63,81].

In this context, the chiller stands out as a critical point for the redistribution of contamination, as the high organic load, simultaneous contact with multiple carcasses, and operational limitations in water renewal may favor the amplification and dissemination of *Salmonella* spp., even under regular hygiene routines [6,23,82].

The clonal persistence observed is consistent with previous reports describing the maintenance of related *Salmonella* spp. genotypes in poultry environments for extended periods, ranging from months to years [4]. Marin et al. [37] reported the persistence of a *Salmonella* Enteritidis isolate in a poultry slaughterhouse even after the implementation of standard cleaning and disinfection protocols, attributing this persistence to the clone’s high resistance to environmental stresses and its ability to recirculate along the processing line.

These findings become particularly relevant when considered alongside the observed phenotypic profiles, as most pulsotypes exhibited multidrug resistance (MDR), ESBL production, and a high capacity for biofilm formation on different surfaces and at different temperatures.

The observation of biofilm production even at lower temperatures, such as 12 °C, in isolates belonging to pulsotypes associated with carcasses intended for the final consumer highlights the risk of *S*. Minnesota persistence under conditions typically considered less favorable for bacterial growth [58,83]. The presence of *Salmonella* spp. on surfaces and equipment represents a key factor for its dissemination within the industrial environment, promoting cross-contamination events during processing and compromising the microbiological safety of the final product [80].

The absence of completely identical profiles, together with the high degree of genetic similarity among variants, suggests the coexistence of microvariations generated under environmental pressure, while maintaining a conserved clonal structure over time [79]. The identification of pulsotypes distributed across the avian intestinal tract, the industrial environment, and the final product reinforces the epidemiological relevance of this serovar and highlights the need for integrated control strategies [20,37,63].

Despite the relevance of the findings presented, several limitations of the present study should be acknowledged. First, the genetic characterization of the isolates was based on targeted molecular approaches, such as conventional PCR, without the use of high-resolution genomic markers, including whole-genome sequencing (WGS) or multilocus sequence typing (MLST). The application of these tools would enable a more accurate assessment of the genetic relatedness among isolates, as well as more robust phylogenetic comparisons with strains circulating in other regions of Brazil and in different international contexts. In addition, the virulence and biofilm associated genes identified in this study were detected exclusively at the genetic level, and no functional or gene expression analyses were performed.

Therefore, the presence of these determinants does not necessarily imply their expression or biological activity under the conditions evaluated. Future studies integrating high-resolution genomic analyses with functional approaches, such as gene expression assays or phenotypic models, are recommended to further elucidate the pathogenic and adaptive potential of the isolates analyzed.

## 5. Conclusions

This study characterized *Salmonella* Minnesota isolates recovered from poultry slaughterhouses using an integrated approach combining genotypic and phenotypic analyses of virulence, antimicrobial resistance, biofilm formation, thermal tolerance, and clonal structure. A high frequency of resistance to clinically relevant antimicrobials was observed, together with a wide distribution of genetic determinants associated with virulence and environmental adaptation.

The isolates exhibited a consistent capacity for biofilm formation, particularly at temperatures compatible with industrial refrigeration environments, as well as distinct responses to thermal exposure, indicating adaptive mechanisms that favor the persistence of *S.* Minnesota throughout processing. PFGE analysis revealed a population structure dominated by genetically related lineages, indicating the circulation and maintenance of genotypes adapted to the industrial environment, with a relevant contribution of the chiller to dissemination dynamics.

Overall, the persistence of *Salmonella* Minnesota in poultry slaughterhouses is sustained by the interplay between antimicrobial resistance, persistence-associated phenotypes, and clonal circulation along the processing chain, representing a significant challenge to food safety and public health. In this context, effective control of *S*. Minnesota requires integrated strategies that address both the mitigation of pathogen introduction from the field and the limitation of its amplification within the industrial environment, with particular attention to critical processing points.

## Figures and Tables

**Table 1:** Phenotypic antimicrobial resistance profiles of *Salmonella* Minnesota isolates from carcasses, cecum, and slaughterhouse chiller water.

Antimicrobial Category	Antimicrobials	Origen			
		Carcass % (n/N)	Cecum % (n/N)	Slaughterhouse Chiller Water % (n/N)	*p*-Value
Amionoglycosides	GEN	42.31 (11/26)	60 (15/25)	45.45 (5/11)	0.566
	N	38.46 (10/26)	32 (8/25)	18.18 (2/11)	0.496
Sulfonamides	SXT	7.69 (2/26)	12 (3/25)	18.18 (2/11)	0.659
Tetracyclines	TET	61.54 (16/26)	60 (15/25)	72.73 (8/11)	0.762
Fluoroquinolones	CP	65.38 (17/26)	52 (15/25)	54.55 (6/11)	0.617
	NOR	7.69 (2/26)	0 (0/25)	0 (0/11)	0.248
	ENR	65.38 (17/26)	68 (17/25)	63.64 (7/11)	0.964
Phenicols	CL	0 (0/26)	8 (2/25)	0 (0/11)	0.224
Fosfomycin	FOS	11.54 (3/26)	0 (0/25)	0 (0/11)	0.116
2nd Generation Cephalosporins	FOX	69.23 (18/26)	56 (14/25)	63.64 (11/11)	0.631
Penicillin	AMP	100 (26/26)	96 (24/25)	90.91 (10/11)	0.357
Carbapenems	ETP	0 (0/26)	0 (0/25)	0 (0/11)	-
β-lactam combination agents	AMC	65.38 (17/26)	60 (15/25)	63.64 (7/11)	0.924
4th Generation Cephalosporins	CPM	42.31 (11/26)	48 (12/25)	36.36 (4/11)	0.807
Monobactams	ATM	57.69 (15/26)	76 (19/25)	63.64 (6/11)	0.390
	CRO	100 (26/26)	92 (23/25)	81.82 (9/11)	0.113
3rd Generation Cephalosporins	CTX	100 (26/26)	96 (24/25)	81.82 (9/11)	0.060
	XNL	69.23 (18/26) ^b^	96 (24/25) ^a^	72.73 (8/11) ^ab^	0.040
	CAZ	100 (26/26)	96 (24/25)	81.82 (9/11)	0.060

GEN: gentamicin; N: Neomycin; SXT: Trimethoprim-sulfamethoxazole; TET: Tetracycline; CP: Ciprofloxacin; NOR: Norfloxacin; ENR: Enrofloxacin; CL: Chloramphenicol; FOS: Fosfomycin; FOX: Cefoxitin; AMP: Ampicillin; ETP: Ertapenem; AMC: Amoxicillin–Clavulanic Acid; CPM: Cefepime; ATM: Aztreonam; CRO: Ceftriaxone; CTX: Cefotaxime; XNL: Ceftiofur; CAZ: Ceftazidime. Values are percentages (%), followed by n/N, where n is the number of resistant isolates and N is the total tested within each origin. Resistance was analyzed as a binary outcome (resistant vs. non-resistant) using generalized linear mixed-effects models (binomial distribution), with sampling origin as a fixed effect and month of sampling as a random effect. When the origin effect was significant, pairwise comparisons were performed using estimated marginal means with Tukey adjustment. Different lowercase letters within a row indicate significant differences among origins (*p* < 0.05). “-” indicates that statistical testing was not applicable due to zero variance.

**Table 2:** Biofilm production profile on polystyrene plates of *Salmonella* Minnesota isolates obtained from carcasses, cecum, and slaughterhouse chiller water.

Biofilm Production Category	Origin			*p*-Value
	Carcass % (n/N)	Cecum % (n/N)	Slaughterhouse Chiller Water % (n/N)	
Non-producer	11.54 (3/26)	4 (1/25)	0 (0/11)	0.357
Weak	15.38 (4/26) ^b^	24 (6/25) ^ab^	54.55 (6/11) ^a^	0.043
Moderate	46.15 (12/26)	56 (14/25)	36.36 (4/11)	0.543
Strong	26.92 (7/26)	16 (4/25)	9.10 (1/11)	0.403

Values are percentages (%), followed by n/N, where n is the number of isolates in each category and N is the total within each origin. For each biofilm category, proportions among origins were compared using binomial models (GLM or GLMM when applicable). When expected cell counts were low, Fisher’s exact test was applied. When significant effects were detected, pairwise comparisons among origins were based on model-estimated marginal proportions with Tukey adjustment. Different lowercase letters within a row indicate *p* < 0.05.

**Table 3:** Biofilm production profile on stainless steel surfaces at different temperatures of *Salmonella* Minnesota isolates obtained from carcasses, cecum, and slaughterhouse chiller water.

Profile	Temperature			Origin				*p*-Value	
	12 °C	16 °C	35 °C	Carcass	Cecum	Slaughterhouse Chiller Water	Temperature	Origin	Interaction
Non adherent % (n/N)	11.29 (7/62) ^b^	9.68 (6/62) ^b^	38.71 (24/62) ^a^	21.79 (17/78)	18.67 (14/75)	18.18 (6/33)	0.000	0.844	0.368
Weak % (n/N)	46.77 (29/62)	62.90 (39/62)	61.29 (38/62)	60.26 (47/78)	52 (39/75)	60.61 (20/33)	0.224	0.529	0.463
Moderate % (n/N)	25.81 (16/62) ^a^	25.81 (16/62) ^a^	0 (0/62) ^b^	11.54 (9/78)	22.67 (17/75)	18.18 (6/33)	0.000	0.162	0.710
Strong % (n/N)	16.13 (10/62) ^a^	1.61 (1/62) ^b^	0 (0/62) ^b^	6.41 (5/78)	6.67 (5/75)	3.03 (1/33)	0.001	0.725	0.855
Samples (n)	62	62	62	78	75	33			

Values are expressed as percentages (%), followed by n/N, where n corresponds to the number of isolates classified in each biofilm production category and N to the total number of isolates analyzed. Biofilm production intensity (non-adherent, weak, moderate, or strong) was analyzed using a mixed-effects ordinal regression model, including temperature, origin, and the temperature × origin interaction as fixed effects, and isolate as a random effect to account for repeated measurements. Global *p*-values for temperature, origin, and their interaction are reported. When significant effects were detected, pairwise comparisons among origins were conducted based on estimated marginal means, with Tukey adjustment for multiple comparisons. Different lowercase letters within the same row indicate statistically significant differences among temperatures (*p* < 0.05).

**Table 4:** Genotypic virulence profile of *Salmonella* Minnesota isolated from carcass, cecum and slaughterhouse chiller water.

Genes	Functional Category		Origin		*p*-Value
		Carcass % (n/N)	Cecum % (n/N)	Slaughterhouse Chiller Water % (n/N)	
*sefA*	Fimbriae	0 (0/26)	0 (0/25)	0 (0/11)	-
*agfA*		100 (26/26)	92 (23/25)	100 (11/11)	0.224
*lpfA*		38.46 (10/26)	44 (11/25)	18.19 (2/11)	0.341
*sipA*	T3SS—Effector proteins	88.46 (23/26)	96 (24/25)	90.91 (10/11)	0.620
*avrA*		100 (26/26)	100 (25/25)	90.91 (10/11)	0.097
*adrA*	Regulatory proteins/biofilm	100 (26/26)	100 (25/25)	100 (11/11)	-
*luxS*		8.06 (5/26)	36 (9/25)	0 (0/11)	0.051
*csgD*		100 (26/26)	100 (25/25)	100 (11/11)	-
*spaN*	T3SS—Structure	92.30 (24/26)	96 (24/25)	90.91 (10/11)	0.357
*invA*	Marker/invasion	100 (26/26)	100 (25/25)	100 (11/11)	-
*tolC*	Intracellular survival/efflux	96.15 (25/26)	76 (19/25)	90.90 (10/11)	0.093

Values are expressed as percentages (%), followed by n/N, where n corresponds to the number of isolates positive for each gene and N to the total number of isolates analyzed per origin. The presence of each virulence gene was analyzed as a binary outcome using logistic regression models, including sampling origin as a fixed effect. Genes showing zero variance (100% or 0% positivity across all origins) were not subjected to statistical analysis and are indicated by “-”.

**Table 5:** Genotypic resistance profile of *Salmonella* Minnesota samples isolated from carcass, cecum and slaughterhouse chiller water.

Genes	Origin			*p*-Value
	Carcass % (n/N)	Cecum % (n/N)	Slaughterhouse Chiller Water % (n/N)	
*qnrS*	3.85 (1/26)	4 (1/25)	0 (0/11)	0.807
*qnrA*	0 (0/26)	0 (0/25)	0 (0/11)	-
*qnrB*	69.23 (18/26)	68 (17/25)	54.55 (6/11)	0.678
*bla*CTX-M-1	15.38 (4/26)	20 (5/25)	18.19 (2/11)	0.914
*bla*CTX-M-2	38.46 (10/26)	52 (13/25)	45.46 (5/11)	0.636
*bla*CTX-M-8	3.85 (1/26)	0 (0/25)	0 (0/11)	0.507
*bla*CTX-M-9	0 (0/26)	0 (0/25)	0 (0/11)	-
*bla*CTX-M-25	0 (0/26)	0 (0/25)	0 (0/11)	-
*fosA3*	0 (0/26)	0 (0/25)	0 (0/11)	-

Values are expressed as percentages (%), followed by n/N, where n corresponds to the number of isolates positive for each resistance gene and N to the total number of isolates analyzed per origin. The presence of each gene was analyzed as a binary outcome using logistic regression models, including sampling origin as a fixed effect. Genes showing zero variance (100% or 0% positivity across all origins) were not subjected to statistical analysis and are indicated by “-”.

**Table 6:** Mean values (log_10_ CFU/mL) of *Salmonella* Minnesota (n = 62) isolates obtained from carcasses, cecum, and slaughterhouse chiller water subjected to different temperatures (4, 10, 37, 50, and 65 °C).

Treatment Temperature/Time	Origin			*p*-Value
	Cecum	Carcass	Slaughterhouse Chiller Water	
4 °C/30 min	9.139 ^ab^	9.084 ^b^	9.247 ^a^	0.034
10 °C/30 min	9.134	9.114	9.175	0.590
37 °C/30 min	9.172	9.130	9.218	0.371
50 °C/3 min	9.123	9.072	9.124	0.550
65 °C/3 min	2.162 ^a^	1.647 ^b^	1.987 ^ab^	0.041

Data are presented as mean values of log_10_ CFU/mL. Comparisons among sampling origins within each temperature–time condition were performed using linear mixed-effects models, including origin and thermal condition as fixed effects and isolate as a random intercept to account for repeated measures. Pairwise comparisons were conducted based on estimated marginal means, with adjustment for multiple comparisons using Tukey’s method. Different lowercase letters within the same row indicate statistically significant differences among origins (*p* < 0.05).

## Data Availability

The data that support the findings of this study are available from the corresponding author upon reasonable request.

## Abbreviations

The following abbreviations are used in this manuscript:

ATCC	American Type Culture Collection
BHI	Brain Heart Infusion
bp	Base pairs
CFU/mL	Colony-Forming Units per milliliter
CLSI	Clinical and Laboratory Standards Institute
ESBL	Extended-Spectrum β-Lactamase
SM	*Salmonella* Minnesota
MDR	Multidrug Resistance
PBS	Phosphate-Buffered Saline
PCR	Polymerase Chain Reaction
PFGE	Pulsed-Field Gel Electrophoresis
SPI	*Salmonella* Pathogenicity Island
Tm	Melting temperature
TSB	Tryptic Soy Broth

## Appendix A

The primer targeting the *adrA* gene was designed in the present study using the Pri-mer-BLAST tool (NCBI; https://www.ncbi.nlm.nih.gov/tools/primer-blast/ (accessed on 22 December 2023)), based on the reference sequence NC_003197.2 obtained from GenBank. The oligonucleotide sequences used in this study are presented in Table A1.

**Table A1:** Primers *adrA* used in this study.

Target Gene	Primer Name	Sequence (5′–3′)	Reference
*adrA*	*adrA* forward	CGTCAATATGTTGCCTGCCG	This study
	*adrA* reverse	AAGAGAGCGTGACTTCCAGC	

The expected amplicon size was 182 base pairs (bp). During the design process, essential physicochemical criteria were considered to ensure amplification efficiency, including melting temperature (Tm) between 58–62 °C, GC content between 45–55%, and absence of undesirable secondary structures such as dimers or hairpins, evaluated using the OligoAnalyzer™ tool (IDT; https://www.idtdna.com/pages/tools/oligoanalyzer) (accessed on 22 December 2023).

PCR reactions were prepared in a final volume of 25 µL, containing 12.5 µL of Go-Taq^®^ Green Master Mix (Promega), 1 µL of each primer (1 µM), 2 µL of template DNA, and 8.5 µL of ultrapure water. The cycling conditions adopted were performed according to De Oliveira et al. [24]. The amplified products were subjected to electrophoresis on 1.5% agarose gels stained with Gel-Red™ (Biotium) and visualized using a transilluminator (Loccus^®^). The *Salmonella* Typhimurium ATCC 14028 strain was used as a positive control for the reaction.
